# Classroom Behavior Recognition Using Computer Vision: A Systematic Review

**DOI:** 10.3390/s25020373

**Published:** 2025-01-10

**Authors:** Qingtang Liu, Xinyu Jiang, Ruyi Jiang

**Affiliations:** 1 Faculty of Artificial Intelligence in Education, Central China Normal University, Wuhan 430079, China; jiangxinyu@mails.ccnu.edu.cn (X.J.); jiangruyi@mails.ccnu.edu.cn (R.J.); 2 Hubei Research Center for Educational Informatization, Central China Normal University, Wuhan 430079, China

**Keywords:** computer vision, offline classroom, teaching behavior, learning behavior, behavior recognition

## Abstract

Behavioral computing based on visual cues has become increasingly important, as it can capture and annotate teachers’ and students’ classroom states on a large scale and in real time. However, there is a lack of consensus on the research status and future trends of computer vision-based classroom behavior recognition. The present study conducted a systematic literature review of 80 peer-reviewed journal articles following the Preferred Reporting Items for Systematic Assessment and Meta-Analysis (PRISMA) guidelines. Three research questions were addressed concerning goal orientation, recognition techniques, and research challenges. Results showed that: (1) computer vision-supported classroom behavior recognition focused on four categories: physical action, learning engagement, attention, and emotion. Physical actions and learning engagement have been the primary recognition targets; (2) behavioral categorizations have been defined in various ways and lack connections to instructional content and events; (3) existing studies have focused on college students, especially in a natural classical classroom; (4) deep learning was the main recognition method, and the YOLO series was applicable for multiple behavioral purposes; (5) moreover, we identified challenges in experimental design, recognition methods, practical applications, and pedagogical research in computer vision. This review will not only inform the recognition and application of computer vision to classroom behavior but also provide insights for future research.

## 1. Introduction

The classroom allows for a series of interactions between teachers and students, and is the main educational space for teachers to help students construct knowledge and develop skills. Analyzing these interactions, that is, classroom behavior analysis, can detect students’ learning status and behavioral characteristics, and provide timely and stage-by-stage feedback to students [1,2]. It also tests teachers’ teaching strategies and promotes teachers’ professional development [3,4]. Classroom behaviors mainly include verbal and nonverbal behaviors between teachers, students, content, and teaching tools. Nonverbal behavior, although frequently ignored, exists consistently throughout the classroom. During the teaching and learning process, information was constantly transmitted between teachers and students through nonverbal behaviors such as physical characteristics, touch behaviors, and spatial factors [5]. Nonverbal behaviors were regarded as an important part of instructional behaviors and had significant potential for helping to better understand the teaching and learning process [6]. Computer vision is a field of artificial intelligence that can derive meaningful information from images, videos, and other visual inputs. By detecting and recognizing nonverbal behaviors (including facial expressions, head movements, body postures, etc.), computer vision has received sustained attention from educational researchers in assessing students’ engagement and teachers’ teaching practices [7,8].

Computer vision uses algorithms and models to learn from a large amount of image or video data and extract useful features, which can be used to recognize and classify objects in the image or video. With the advancement of deep learning and the development of acquisition and hardware devices, computer vision using non-intrusive devices such as high-definition surveillance cameras can achieve large-scale concomitant acquisition and automated intelligent annotation of classroom behaviors. Some studies have attempted preliminary applications in real classrooms [9,10]. Traditional manual coding methods are prone to inconsistencies in evaluation results and ambiguities in the evaluation process, due to individual differences or empirical tendencies. It is also difficult to handle large-scale data due to limited physical strength and energy. Computer vision methods have high accuracy and reliability, which can reduce human bias and provide more objective analysis results. Their high-speed computing power enables real-time classroom monitoring. Moreover, these fine-grained recognition results can provide a scientific basis for educational decision-making. In the education field, existing studies have often used computer vision to characterize the emotional, attentional, and engagement states of teachers and students by detecting nonverbal behavioral features such as facial expressions, head gestures, and body gestures [11,12,13].

Among them, facial expressions are a common emotion recognition mechanism. Facial expressions were valuable in characterizing subject experience, cognitive processes, and behavioral influences on emotions [14]. In addition, facial expressions are rich in immediate interactive information. Teachers have adjusted their instructional methods by observing the student’s learning status based on facial expressions [15,16]. Facial expression recognition based on computer vision was frequently used to continuously characterize teachers’ and students’ classroom emotions and engagement states [17,18]. A review study focusing on computer vision for facial expression recognition is detailed in two categories: traditional machine learning and deep learning [19].

Attention is essential for learning. Computer vision for body movement feature recognition was an important approach to attention measurement and tracking [20]. Computer vision recognizes teachers’ and students’ gaze direction and head and body movements through posture estimation and face detection. This could automatically assess whether students are following the teacher or the content, but also the teachers’ attention span [2,21].

In the classroom, students’ discourse information is limited. Body movements could directly and effectively reflect the students’ individual interaction level and engagement status [1]. Computer vision could recognize body postures from body, head, and hand gestures, such as hand raising and reading [22,23]. In addition, some studies used deep learning methods to automatically extract relevant features to assess learners’ cognitive engagement [24,25].

However, real classroom contexts are complex, with multiple and intertwined factors influencing changes in teacher and student status. More and more researchers have found the limitations of using unimodality to characterize behavioral states. Existing studies have shifted from unimodal computing to multimodal fusion, using multiple modal data (video data, audio data, and physiological data) to jointly characterize teacher and student status [26,27]. Second, there has been a shift from focusing on single behaviors themselves to emphasizing the connection between behaviors and events, enhancing the educational phenomena’s interpretability [28,29]. Meanwhile, the continuous optimization and improvement of deep neural networks have significantly improved performance in image recognition and processing tasks, which has greatly contributed to the development of computer vision [30,31].

Despite the increasingly extensive research on computer vision for classroom behavior recognition, existing studies have not reached a basic consensus on behavioral characterization or recognition techniques. Relevant reviews are shown in Table 1. Jiang and Fu [7] introduced deep learning-based target detection methods and pose and face-oriented feature extraction and classification on a technical level, but lacked an overview of the review content. Dewan, Murshed, and Lin [8] highlighted computer vision-based engagement detection for online learning. However, detection objects, datasets, and evaluation methods were provided, and specific behavioral indicators and identification techniques were ignored. Karimah and Hasegawa [32] reviewed automatic engagement estimation methods, detailing the processing steps and estimation methods of both machine learning and deep learning methods, but lacked an analysis of the correlation between behavioral indicators and recognition methods. Existing review studies concentrate on a single recognition goal while focusing on the description of recognition methods. However, this study emphasizes recognition goals and their classification in real classroom scenes, specific recognition methods, and challenges existing research faces. Therefore, this study aims to summarize previous experiences and identify future directions in terms of goal orientation, recognition techniques, and research challenges, which will help subsequent studies to refine the indicator construction and optimize the algorithm selection. The following research questions guided our study:

- RQ1. What is the research purpose, educational context, and classroom environment of the reviewed studies?
- RQ2. How did the reviewed studies use computer vision techniques for behavioral recognition?
- RQ3. What are the challenges and limitations of the reviewed studies?

## 2. Methods

### 2.1. Search Strategy

Given that computer vision-based classroom behavior recognition covered multiple related research fields, including education, information science, and engineering, we searched six popular databases, including Web of Science, Springer Link, ScienceDirect, IEEE Explore, ACM Digital-Library, and ERIC. This study used the query string “classroom AND computer vision AND (student engagement OR student behavior OR teacher behavior) AND (recognition OR detection OR identification OR classification)” for a parallel search. All searches were limited to title, abstract, and keywords.

### 2.2. Study Selection Process

**Figure 1:**
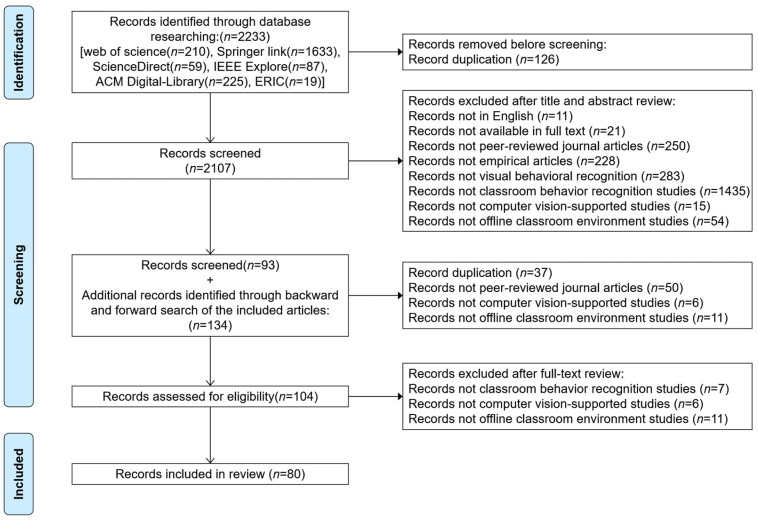
The systematic literature review procedures.

This study followed procedures in the Preferred Reporting Items for Systematic Reviews and Meta-Analyses (PRISMA) statement [33], as shown in Figure 1. The search period was from 16 October to 23 October 2024, and a total of 2233 articles were collected. First, 126 duplicate articles were excluded, and the remaining 2107 articles were randomly assigned to two authors for independent review. In the screening process, we first considered behavior recognition in real classrooms. Second, behavior recognition methods were supported by computer vision. Third, to ensure the representativeness of the research articles, only peer-reviewed journal articles were considered. Finally, we focused on papers written in English. Therefore, the articles’ titles, abstracts, and keywords were analyzed according to the inclusion and exclusion criteria shown in Table 2. For divergent articles, the two researchers held a meeting to discuss whether they met the inclusion or exclusion criteria. After the first phase of screening, 93 articles remained. Then, we performed citation tracking to ensure complete coverage, retrieving a total of 134 additional articles. After screening for inclusion and exclusion as described above, a total of 104 articles remained, which were read in full and screened using the same methodology, and 80 articles were identified for data extraction.

### 2.3. Data Extraction and Coding

In this study, Endnote and MS Excel were used to extract the required data from the literature review and code. According to the research questions, data extraction and coding were divided into four sections, as shown in Table 3. Through Cohen’s kappa analysis, the inter-rater reliability was 0.91. Finally, a meeting was held to negotiate an agreement on inconsistent coding results, thus completing the coding procedure.

## 3. Results

**Figure 2:**
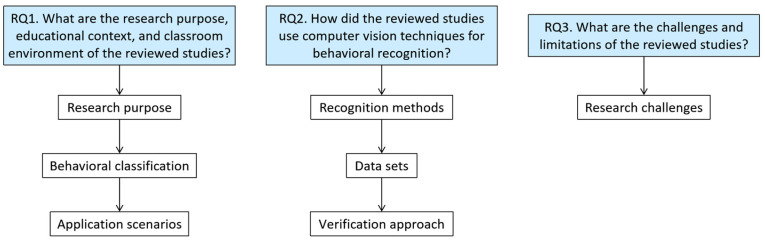
The analysis process of present work.

The present section details the methodology used to answer the research questions of this study. Figure 2 presents the sequence of steps in analyzing the results, which is described step-by-step in the following subsections.

### 3.1. What Are the Recognition Goals, Educational Context, and Teaching Environment of the Reviewed Studies?

**Figure 3:**
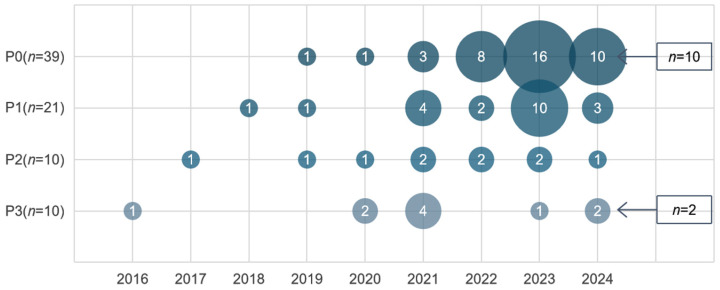
Distribution of recognition purposes and publication years.

The recognition purposes of the reviewed studies were summarized into four categories: (P0) physical action was the most frequent, followed by (P1) learning engagement, (P2) attention, and (P3) emotion. Figure 3 shows the four recognition purposes’ distribution in publication years. Computer vision for classroom behavior recognition has increased significantly from 2020 to 2024. Between 2016 and 2018, the reviewed studies focused on learning engagement, attention, and emotion. Physical action recognition began to accumulate after 2019, until it peaked in 2023.

Table 4 presents the behavioral classifications of the research purpose, with detailed classifications and sources in Appendix A: (1) Physical action recognition focused on the simple behavioral actions of teachers and students, with student behaviors being the most frequent (28/39), followed by teacher behaviors (6/39) and teacher-student behaviors (5/39). Furthermore, Yin Albert et al. [34] and Ye et al. [35] subdivided the physical actions into positive and negative states. In addition, Wu [36] focused on the students’ abnormal behavior detection, including aggressive and non-aggressive behaviors. (2) In learning engagement recognition, most of the studies focused on the overall level of students’ learning engagement (13/21) and the subcomponents of learning engagement (8/16), including behavioral, emotional, and cognitive engagement. Moreover, Yi [37] measured students’ learning interests based on three dimensions: cognitive attention (Attention), learning emotion (Emotion), and thinking activity (Thinking). (3) Attention recognition considered attention levels (7/10) and attention directions (3/10). Attention directions included looking at the teacher, looking at the board, looking at notes, and other directions [2,38]. (4) In emotion recognition, studies mainly focused on students’ classroom emotions (7/10) and only one study focused on teachers’ classroom emotions [39]. In addition, Behera, Matthew, Keidel, Vangorp, Fang, and Canning [29] and Lee and Lee [28] reflected on the difficulty and type of learning tasks based on the students’ facial features upon answering the questions.

The distribution of educational contexts, learning environments, and collection devices in the reviewed studies is shown in Table 5. First, most of the studies chose higher education (*n* = 48), followed by k-12 (*n* = 26), and three studies chose a multi-segment educational context [18,40,41], while three studies did not specifically mention it. Second, constrained environments are those in which the subjects will be instructed by the researcher to exhibit classroom interaction behaviors, and natural environments without the intervention. Overall, most of the studies were conducted in natural environments (*n* = 70), especially the classical classroom (*n* = 52). Finally, most studies collected data via cameras (*n* = 71), followed by computer webcams (*n* = 6).

### 3.2. How Did the Reviewed Studies Use Computer Vision Techniques for Behavioral Recognition?

**Figure 4:**
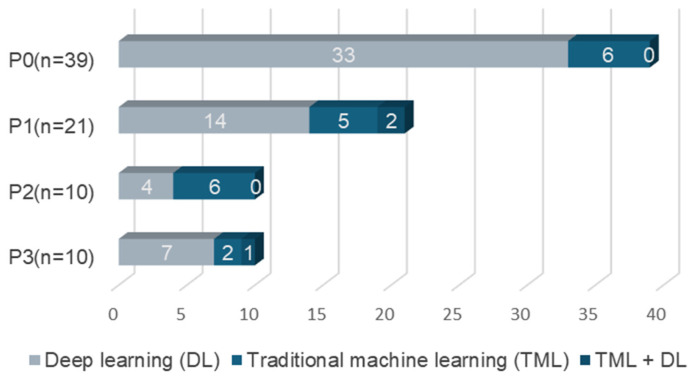
Recognition purposes and recognition methods.

According to feature extraction, computer vision can be categorized into two types: traditional machine learning methods and deep learning methods. Traditional machine learning methods require manual design and the extraction of features, which are then used to train models such as support vector machines or decision trees. This method relies on human experience and prior knowledge to define classification rules and feature selection. The deep learning method can abstract and represent input data at multiple levels by constructing a multi-layered neural network structure, which can automatically learn complex patterns and feature representations in the data. It avoids the complexity of manually crafting features, thus simplifying the processing flow and improving the model’s recognition performance. Figure 4 shows the computer vision methods used for each research purpose. The results revealed that the review studies mainly used deep learning (DL) (*n* = 58) and traditional machine learning (TML) (*n* = 19). In addition, three studies used traditional machine learning and deep learning for different behavioral feature extraction.

Feature extraction types can be categorized into geometric features, texture features, motion features, spatial–temporal features, and deep features based on feature content and usage. Table 6 shows the types and contents of features extracted in the traditional machine learning method and deep learning method. Traditional machine learning methods mainly extract geometric features (*n* = 20), followed by texture features (*n* = 6), and finally, motion features (*n* = 4). Deep learning methods mainly detected deep features (*n* = 45). Eight studies additionally detected surrounding small objects, utilizing the complementary nature of different relational features to enhance recognition performance [42,43]. Moreover, five studies improved the accuracy and robustness of behavior recognition by capturing spatial and temporal information in video frames [44,45]. Finally, only two studies specifically indicated the use of texture features and motion features [46].

Table 7 shows the three classifier types used by TML, including shallow classifiers, deep classifiers, and joint classifiers. Shallow classifiers rely on manually selected features through models with only one or a few layers of nonlinear processing units. The reviewed studies mainly used formula rule definition (*n* = 7), support vector machines (SVM) (*n* = 6), random forests (RF) (*n* = 5), decision trees (DT) (*n* = 3), and Bayesian classifiers (*n* = 2). Compared to shallow classifiers, deep classifiers can automatically learn features through a multilayer nonlinear transformation and achieve significant performance improvement in image classification. One study used VGG16 for classification [22]. In addition, Gu and Li [47] combined the features of the two classifiers, utilized the multilayer structure of BP neural networks for feature extraction, and combined the powerful classification capability of SVM to improve the accuracy.

Table 8 shows the DL corresponding to recognition purposes. Physical action recognition mainly used the YOLO series (*n* = 12) and CNN (*n* = 7) for feature extraction and behavior classification. Learning engagement recognition used various approaches such as the YOLO series (*n* = 4), CNN (*n* = 4), ResNet framework (*n* = 3), Transformer framework (*n* = 2), and VGG framework (*n* = 2). Emotion recognition mainly used the CNN framework (*n* = 4). Overall, the YOLO series was the most widely used model in DL (*n* = 9).

**Figure 5:**
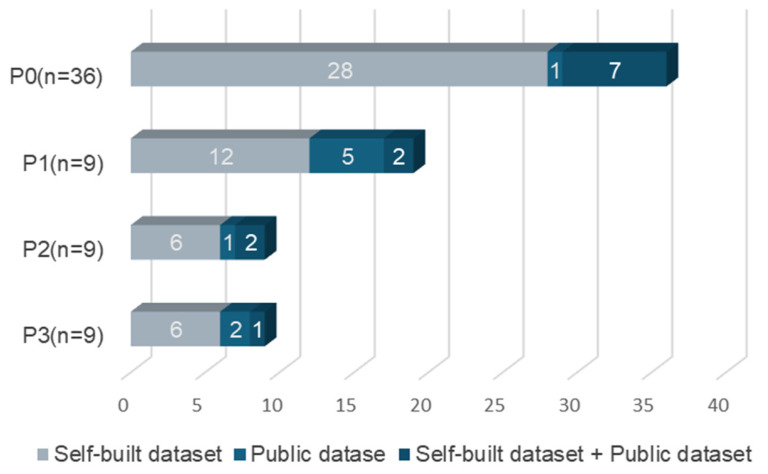
Recognition purposes and dataset construction.

Figure 5 shows the construction method of the training dataset in the reviewed studies. Fifty-two studies chose a self-built dataset, nine studies chose a public dataset, twelve studies combined a self-built dataset and public dataset training data, and seven studies did not specify. Regarding the sample size of the self-built dataset, twenty-five studies had a sample size of less than 10,000, twenty-three studies had a sample size between 10,000 and 100,000, and three studies had a sample size of more than 100,000. Eleven studies did not specify.

Table 9 shows the five verification approaches used in the reviewed studies. Model metrics assessment was the most frequent (*n* = 75), using mainly accuracy, recall, F1 score, and confusion matrices, followed by comparison with other methods (*n* = 46), public dataset testing (*n* = 12), correlation analysis with human labeling and self-report (*n* = 10), and feedback from teacher and student interviews (*n* = 1). Bao [48] compared students’ emotional learning engagement (ELE), as measured by the multiscale perception network (MP-FERS), with academic records and self-reported ELE. The results showed that students’ ELE, as measured by MP-FERS, was a significant predictor of academic performance and was more reflective of true learning status than self-reported ELE. Compared to self-reporting, which is a subjective judgmental assessment, computer vision-based behavioral recognition is a more objective measure that is not easily biased by students’ intentions.

### 3.3. What Are the Challenges and Limitations of the Reviewed Studies?

The challenges and limitations discussed in the reviewed studies were summarized into four categories, as shown in Table 10. Firstly, regarding the experimental process’ limitations, most studies mentioned the occlusion, lighting, and multi-scale issues caused by the classroom environment complexity (*n* = 19), and the limited training dataset of the classroom videos (*n* = 14). Second, regarding recognition methods, twenty-two studies pointed out that more features should be added to improve the effectiveness of behavioral recognition. Third, for practical applications, most studies emphasized generalization, including demographics, different application scenarios, and temporal generalization (*n* = 15), as well as the impact of model complexity and computer computing power on real-time performance (*n* = 13). Finally, seven studies suggested the need to further explore the relationship between behavior and performance.

## 4. Discussion and Conclusions

### 4.1. Goal Orientation

From the reviewed studies, we observed four recognition purposes including physical action, learning engagement, attention, and emotion. Among them, physical action recognition and learning engagement recognition are the two main research themes.

Physical action recognition was mainly focused on recognizing the simple external behaviors of teachers and students, for example, teachers’ guiding, boarding, and walking, and students’ raising hands, reading, taking notes, sleeping, and playing on cell phones [49,50]. However, we found that these behavioral classifications could not fully cover the behaviors occurring in the classroom. Moreover, these behaviors were often independent and difficult to reflect the realities of the classroom, such as instructional events and activities. Students’ learning behaviors are closely related to teaching and learning activities. Zhao, Li, and Jia [1] constructed a behavioral engagement model framework to measure student engagement by uniting teacher and student action behaviors, and the results demonstrated that classifiers using teacher behaviors more accurately predicted student learning engagement. In light of this, it is recommended to include instructional activities in the recognition objectives to improve the reliability of the results.

Learning engagement recognition focused on the overall level and subcomponents of students’ learning engagement. We found that most studies categorized the overall level of learning engagement into multiple levels [51,52], and identified disengaged students through colored labels. Some studies have conceptualized learning engagement as a multidimensional construct measuring cognitive engagement, affective engagement, and behavioral engagement. Mayer [53] suggested that it is cognitive activities rather than behavioral activities that facilitate constructivist learning. Some observational studies have shown that it is possible to infer students’ cognitive engagement from their behavior [54]. Among the reviewed studies, the ICAP framework was used as the theoretical basis to assess students’ cognitive engagement based on external behaviors in terms of four dimensions: passive, active, constructive, and interactive [24,55,56]. Notably, such internal behavioral labeling needs to be combined with students’ own reports to ensure the reliability of behavioral labeling.

Attention recognition was concerned with the level and direction of attention. We observed that some studies concerned teachers’ and students’ eye gaze or head and body orientation in order to track attention content, including whether facing the board, notes, teacher, or students [2,38]. Other studies detected concentration or distraction from facial features and body gestures [57,58]. Attentional mechanisms involve how to select, regulate, and maintain attention to the information most relevant to behavior [59]. Selection is a core function of attention. However, Keller et al. [60] argued that averting one’s gaze from the instructor or lecture slides may indicate a shift in attention (e.g., from external to internal attention) rather than a distraction. Thus, a simple categorization may overlook students’ active processing. We suggest that future studies can further refine the categorization of attention in the classroom.

Emotion recognition was primarily concerned with students’ learning emotions, with facial features being the main visual cues [17,18]. There are various emotional experiences that students feel in learning and teaching situations [61]. Many studies have shown that understanding student emotions can help improve academic performance and learning performance [62]. However, teachers’ emotion recognition is lacking. Frenzel et al. [63] suggested that teachers’ emotions affect students’ learning behavior in at least three ways: direct transmission effects, teacher–student relationships, and nonverbal social messages. In this context, it is recommended that future studies focus on teachers’ emotional expression characteristics and behavioral interpretations. Based on the basic emotion theory, Keltner et al. [64] argued that the multimodal and dynamic behavioral patterns of emotion expression include not only facial muscle movements, but also body movements, gestures, and vocal cues. Integrating multiple modalities for emotion recognition has become a future trend [26]. However, large-scale and multimodal emotion datasets are still scarce.

Overall, we found that behavioral categorization definitions are diverse and coarse-grained for the same recognition goal. Meanwhile, most studies lacked interaction with the content and environment. Therefore, future studies should pay more attention to combining teaching theory and teaching practice, constructing scientific, standardized, and easy-to-quantify indicators, and improving both the interpretability of recognition results and the applicability of classroom scenarios. Meanwhile, multimodal analysis, encompassing the fusion of visual, audio, and sensor data, enables a more comprehensive understanding of classroom interactions [65]. For example, Zou et al. [66] combined information from facial expressions, speech patterns, and physiological signals, which has been used to improve the accuracy and robustness of emotion recognition. Furthermore, few studies focused on teachers’ nonverbal behavior. This may be related to the size of the dataset. The dataset for teacher behavior recognition requires a large number of classroom videos to cover different types of objects and behaviors. The teacher is the key player in classroom implementation, and their behaviors directly affect student learning engagement and classroom quality [67,68]. Therefore, future studies are encouraged to continue in the current direction, while strengthening teachers’ behavioral recognition.

In terms of classroom environment, we found that the reviewed studies mainly focused on natural classrooms, especially for students in higher education, followed by k-12. Some studies have suggested that constrained environments affect the real performance of teachers and students [23]. Therefore, natural environments could be more useful for observing classroom realities and assessing model effectiveness in practical applications. It is worth noting that the k-12 students’ learning process occurs more in the classroom than with college students [69]. From this perspective, exploring the characteristics of classroom learning behaviors plays an important role in enhancing the learning performance of k-12 students.

### 4.2. Recognition Methods

The reviewed studies used traditional machine learning methods and deep learning methods to recognize behaviors of visual cues, with the deep learning method used more frequently. A possible explanation is that deep learning has powerful feature learning capabilities, with better recognition performance and faster detection.

Traditional machine learning extracted geometric features, texture features, and motion features. These features mainly included human keypoints, facial feature points, AUs, and the freedom degrees of the head in all three directions. Notably, due to the reliance on hand-crafted features, there may be cases where inadequate feature selection results in the failure to provide accurate results [57]. Especially for behavior recognition and the classification of complex scenes, as the number of distinguished categories increases, it can make the feature selection and model tuning process very cumbersome, or even difficult to achieve. Therefore, we recommend selecting and extracting features based on specific application scenarios and behavior recognition tasks to ensure that they can effectively capture behavior-related information.

Deep learning also increases the recognition of complex teaching behaviors through improved ROI detection methods and 3D-CNN enhanced feature representation. Deep learning methods contain a variety of models such as the YOLO series, CNN, ResNet framework, and Transformer framework. We found that the YOLO series was used most frequently for multi-class recognition purposes. Compared to other models, the YOLO series has the ability to detect objects quickly and accurately by solving the detection task as a regression problem and directly predicting the object’s category and location in a single forward propagation [70]. Future research can explore how to integrate different deep learning models to fully utilize the advantages of each model and achieve complementary strengths. For example, by combining the YOLO model for target detection and 3D-CNN for temporal–spatial feature extraction, the ability to recognize student behavior in complex dynamic scenes can be improved [71]. In addition, the feature learning of deep learning is a black box, and it is difficult to explain its decision-making process. Some studies have attempted to make the model’s decision-making process more transparent by introducing attention mechanisms and visualization techniques [72,73].

Regarding the dataset selection, all studies opted for self-built training datasets. In order to ensure the effectiveness of feature extraction and the recognition results, it is necessary to establish the corresponding dataset. Because of the scarcity of classroom videos, public datasets were used for transfer learning. This reduces the lengthy training process of self-built datasets by using pre-training weights. Smaller datasets would affect the reliability and relevance of the results, especially for deep learning methods. We call on future studies using large-scale datasets for training models to meet the generalization capabilities of demographics and different application scenarios.

Among the reviewed studies, model metrics assessment and comparison with other methods were the two main verification approaches. However, both methods were conducted based on the reliability of artificially labeled datasets. Studies have shown that human-labeled data may be unreliable [24,74], especially on the internal states of teachers and students, such as thinking and wandering. Therefore, future studies can strengthen the correlation analysis between model predictions and self-reports of teachers and students, as well as improve the model through feedback from teacher and student interviews.

### 4.3. Research Challenges

We summarized the challenges and limitations indicated by the reviewed studies in four aspects.

For the experimental design aspect, the reviewed studies emphasized the impact of complex classroom environments on recognition effectiveness, such as occlusion, light, and limited camera resolution. Future studies could emphasize improved solutions for behavior recognition under adverse conditions such as occlusion and light. In addition, future studies could expand the sample size of classroom videos and extend the experimental duration to enrich and balance the dataset distribution. Generative large language models generate high-quality image and video data from text, images, and video, to create synthetic data that simulate real classroom behavior. For example, Lin et al. [75] used Stable Diffusion and the DALL-E 3 model to generate images of students focusing, raising their hands, writing, sleeping, and using their cell phones in the classroom, enhancing the depth and breadth of the dataset.

For recognition methods, most studies emphasized the consideration of more visual cues, speech cues, and physiological signals to enhance the effectiveness of recognition results [29,76,77]. Meanwhile, the coding rules for multiple and similar postures are important, especially for behavioral recognition based on human skeleton data, where irregular postures can easily affect recognition [78]. Some studies have only detected some of the subcomponents of learning engagement [24,79,80], and there is a need to detect more comprehensive dimensions in the future. In addition, additional external verification can help compensate for the incomplete reliability of human-labeled data, such as teacher judgment and student learning outcomes.

For the model application aspect, most studies have highlighted the need for more group and environmental testing to enhance the generalization of recognition models [49,81,82]. To achieve large-scale detection and real-time annotation of recognition methods in the classroom, future research is encouraged to develop lightweight models that increase computational efficiency while maintaining high detection accuracy. In addition, privacy issues of data collection and processing need to be considered in the future.

For the practical application, some studies proposed to enhance the scalability of the recognition model, for example, the deployment of servers and the visualization of feedback data to enhance practical application in the classroom. This could help teachers monitor and evaluate their teaching behaviors in real time, so that they can optimize their teaching methods and improve classroom interactions to better meet the learning needs of their students. For example, Huang [24] pushed adaptive learning materials to learners by measuring and calculating their engagement level. In addition, the recognition model could be embedded in educational application devices. For example, educational robots could improve the quality of interactions by recognizing learners’ behaviors.

For the pedagogical research aspect, students’ classroom learning participation is considered to be a precondition for achieving good learning outcomes, which is positively correlated with a higher level of competence development [83]. Among the reviewed studies, most studies still remain on the models and methods of behavior recognition. More studies are needed to test the practical application effect of recognition methods and to explore the characteristics of classroom teaching and learning behaviors in the future, as well as the influential mechanism research on the relevant stakeholders’ usage feelings and feedback contents. These findings will facilitate teaching administrators to more accurately assess the quality of teaching and improve teachers’ teaching evaluation and training. In addition, these findings will help to identify and address educational disparities between different regions or schools and promote a balanced distribution of educational resources.

In summary, the findings in this study provide a new understanding of visual cue-based computer vision recognition methods in classroom teaching and learning behavior recognition. We hope that the results of the study will provide a basis for educational researchers to find new recognition indicators and recognition methods in this field. However, there are some limitations. In terms of search strategy, we only reviewed journal publications from 2016 to 2024, which is not representative of all research trends. Therefore, it is recommended that a large-scale review be conducted in future to gain a broader understanding of visual cue-based behavior recognition in the classroom.

## Figures and Tables

**Table 1:** Comparison with previous reviews. (✓: Discussed, ✗: Not Discussed).

Authors	Focus	RQ1. Behavioral Classification	RQ1. Application Scenarios	RQ2. Recognition Methods	RQ2. Datasets	RQ2. Verification Approaches	RQ3. Research Challenges
Jiang and Fu [7]	Student classroom behaviors	✓	✗	✓	✗	✗	✗
Dewan, Murshed, and Lin [8]	Engagement in online learning	✗	✗	✓	✓	✓	✗
Karimah and Hasegawa [32]	Engagement in smart education/learning settings	✓	✗	✓	✓	✓	✗
Our Work	Classroom behaviors	✓	✓	✓	✓	✓	✓

**Table 2:** Inclusion and exclusion criteria.

Inclusion Criteria	Exclusion Criteria
Articles written in English.	Duplicate studies.
Peer-reviewed journal articles.	Unavailability of full text.
Articles on behavior recognition in the classroom.	Non-journal papers, such as conference papers, journal presentations, book manuscripts, and reports.
Articles on behavior recognition based on computer vision support.	Studies are not to be reviews, meta-analyses, or review articles.
Articles on offline classroom environments.	Articles are not relevant to the research topic.

**Table 3:** The coding schemes.

Analysis Dimension	Extracted Data
Goal Orientation	Research purpose, behavioral classification, educational context, classroom environment, and collection equipment.
Recognition Techniques	Methods (feature extraction, classifiers), sample size, and verification approach.
Research Challenges	Challenges.

**Table 4:** Research purposes and behavioral classifications.

Research Purpose	Behavioral Classification	Number
Physical action (*n* = 39)	Student behaviors	28
	Teacher behaviors and student behaviors	6
	Teacher behaviors	5
Learning engagement (*n* = 21)	Learning engagement levels	13
	Cognitive engagement	6
	Behavioral and emotional engagement	2
Attention (*n* = 10)	Attention levels	7
	Attention direction	3
Emotion (*n* = 10)	Student emotions	7
	Teacher emotions	1
	Learning task types	2

**Table 5:** Distribution of educational contexts, learning environments, and collection devices.

Educational Context	Learning Environment		Collection Device
Higher education (*n* = 48)	natural environment	College classroom (*n* = 29), smart classroom (*n* = 5), intelligent classroom (*n* = 3), computer lab (*n* = 2), college seminar (*n* = 1)	Camera (*n* = 37), computer’s webcam (*n* = 1), Kinect infrared camera (*n* = 1), camera and computer’s webcam (*n* = 1)
	constrained environment	Computer-based learning (*n* = 3), college classroom (*n* = 2), lab (*n* = 2), intelligent classroom (*n* = 1)	Camera (*n* = 5), computer’s webcam (*n* = 3)
k-12 (*n* = 26)	natural environment	Classical classroom (*n* = 22), computer lab (*n* = 1), blended synchronous classroom (*n* = 1), intelligent classroom (*n* = 1), smart classroom (*n* = 1)	Camera (*n* = 24), computer’s webcam (*n* = 1), smartphone camera (*n* = 1)
k-12, Higher education (*n* = 2)	natural environment	Classical classroom (*n* = 1)	Camera (*n* = 1)
	constrained environment	Computer-based learning (*n* = 1)	Computer’s webcam (*n* = 1)
Early childhood education, Higher education (*n* = 1)	natural environment	Classical classroom (*n* = 1), college seminar (*n* = 1)	Camera (*n* = 1)
Not specified (*n* = 3)	natural environment	Classical classroom (*n* = 2)	Camera (*n* = 2)
	constrained environment	Lab (*n* = 1)	Camera (*n* = 1)

**Table 6:** Feature extraction methods and feature extraction types.

Feature Extraction Method	Feature Extraction Type	Description
Traditional Machine Learning Method	Geometric features (*n* = 20)	Body keypoints, facial feature AU, facial feature coordinates, head pose (X, Y, Z), 3D position estimation, histogram of orientation gradients (HOG)
	Texture features (*n* = 6)	Face texture, local binary pattern (LBP), local directional pattern (LDP), scale invariant local ternary pattern (SILTP), Gabor, image intensity
	Motion features (*n* = 4)	Maximum, median, standard deviation, velocity of body movement
Deep Learning Method	Depth features (*n* = 45)	ROI and surrounding small object detection
	Geometric features (*n* = 10)	Body keypoints, facial feature coordinates, head pose (X, Y, Z)
	Spatial–temporal features (*n* = 5)	Spatial and temporal dimension information in video data
	Texture features (*n* = 1)	RGB image features, data-enhanced features, lab color vectors
	Motion features (*n* = 1)	Optical flow features

**Table 7:** Classifiers in traditional machine learning methods.

Classifier Type	Description
Shallow classifier	Formula rule definition (*n* = 7), support vector machine (*n* = 6), random forest (*n* = 5), decision trees (*n* = 3), Bayesian classifiers (*n* = 2), KNN (*n* = 1), logistic regression (*n* = 1), k-means (*n* = 1)
Deep classifier	VGG16 (*n* = 1)
Joint classifier	BP neural networks and SVM (*n* = 1)

**Table 8:** Recognition purposes and deep learning methods.

Basic Framework	Physical Action (*n* = 33)	Learning Engagement (*n* = 16)	Attention (*n* = 4)	Emotion (*n* = 8)
YOLO series (*n* = 18)	YOLOv5 (*n* = 3), YOLOv4 (*n* = 2), YOLOv3 (*n* = 1), YOLOv5s (*n* = 1), YOLOv7 (*n* = 1), YOLOv7-tiny (*n* = 1), YOLOv8 (*n* = 1), YOLOv8n (*n* = 1), YOLOv8s (*n* = 1)	YOLOv8n (*n* = 3), YOLOR (*n* = 1)	YOLOv5 (*n* = 2)	
CNN (*n* = 15)	CNN (*n* = 2), BP-TBR (*n* = 1), 3D-CNN (*n* = 1), AIA network (*n* = 1), BCNN (*n* = 1), CBPH-Net (*n* = 1)	CNN (*n* = 1), Light Fer (*n* = 1), MobileNetV2 (*n* = 1), CoAtNet (*n* = 1)		CNN (*n* = 3), EHMFCNN (*n* = 1)
ResNet framework (*n* = 9)	ResNet-101 (*n* = 1), SlowFast (*n* = 1), STAR-3D (*n* = 1)	ResNet-50 (*n* = 2), Inception-ResNet-V2 (*n* = 1)	RHG-Net (*n* = 1)	ResNet-50 (*n* = 2)
Transformer framework (*n* = 7)	Swin Transformer (*n* = 2), RT DETR (*n* = 1)	CNN-Transformer (*n* = 1), ResNet-Transformer (*n* = 1)	Vision Transformer (*n* = 1)	MLGPN (*n* = 1)
DNN (*n* = 7)	DNN (*n* = 2), BiLSTM-AT (*n* = 1), GCN (*n* = 1), MoGRU (*n* = 1)	DNN (*n* = 1)		DNN (*n* = 1)
VGG framework (*n* = 3)	VGG16 (*n* = 1)	VGG16 (*n* = 2)		
SSD framework (*n* = 3)	SSD (*n* = 2)	Mobilenet-SSD (*n* = 1)		

**Table 9:** Verification approaches.

Verification Approach	Number
Model metrics assessment	75
Comparison with other methods	46
Correlation analysis with human labeling and self-reporting	12
Public dataset testing	10
Feedback from teacher and student interviews	1

**Table 10:** Research challenges and limitations.

Categories	Challenges and Limitations	Number
Experimentation	Occlusion, lighting, and multi-scale problems	19
	Limited training dataset	14
	Motion blur and image quality (limited camera resolution)	9
	Unequal dataset distribution	5
	Short experimental time	1
Method	More features should be added to improve behavioral recognition performance	22
	More comprehensive dimensions were not characterized	10
	Influence of constrained environment on student behavior	5
	Classification and coding rules to cope with multiple and similar postures	3
	Influence of individual student characteristics on student behavior	2
	Lack of additional external verification	2
	Reliability of human-labeled datasets	1
Application	Generalization ability across demographics, different application scenarios, and time	15
	Limitations on training time due to model complexity and computer computing power	13
	Deployment of servers or embedded devices	8
	Privacy issues in data collection and processing	5
	Visual extensions of feedback data	4
	High cost of system usage and maintenance	2
Pedagogy	Exploring the relationship between behavior and performance	7

## Data Availability

Data will be made available on request.

## Appendix A

**Research Purpose**	**Behavioral Classification**	**Description**	**Source**
Physical action (*n* = 39)	Student behaviors	Sitting, raising hand, lowering head	[84]
		Discussion, engaged, sleeping, eating, using smart phone	[82]
		Asking, boring, bowing, looking	[85]
		Bend, jack, jump, walk, wave1, wave2	[47]
		Aggressive, non-aggressive	[36]
		Sleeping, playing on phone, moving, eating, reading, writing, using computer	[86]
		Raising their hands, bending over, walking back forth, writing on the blackboard, looking up, bowing their heads, standing, raised hands, lying on their desks	[23]
		Listening, looking down, lying down, standing	[87]
		Playing on cell phones, sitting with hands on face, sitting turned right, sitting turned left, bowing, sleeping, drinking water, standing, yawning, having class, unknown behaviors	[88]
		Positive behaviors: standing and listening; neutral behaviors: head-down; negative behaviors: head-turning	[34]
		Raise hand, sleep, stand, use phone, take note, listen to class	[22]
		Hand-raising	[89]
		Reading or writing, looking at the blackboard, playing with cellphone, looking around, standing up, raising hand, lying down	[90]
		Write, read, look up, turn head, raise hand, stand, discuss	[77]
		Listening, looking around, lying on the table, reading, writing, using cell phone, talking	[71]
		Look at phone, listen, stand, sleep, sit, talk, write	[74]
		Sitting, standing, raising hand, average	[91]
		Looking to the right, looking left, playing with cell phones, sleeping, standing, speaking	[92]
		Raising hands, reading, writing	[93]
		Write, read, look up, turn head, raise hand, stand, discuss	[73]
		Looking at the board, looking down, turning head/turning around, talking, standing up, raising hands, lying on the table	[94]
		Raising hand, standing up, writing, slippage, listening	[95]
		Focusing, raising hands, writing, sleeping, using phones	[79]
		Hand-raising, interacting, sitting, turning around, writing	[46]
		Listen to lecture, play on phone, raise hand, write, sleep	[43]
		Raising hand, reading, writing	[41]
		Read, take notes, talk, tidy hair, rest head on hand, lie on desk, listen, raise a hand, stand, use the phone	[44]
		Raising their hands, reading, writing, playing on their cell phones, looking down, leaning on the table	[72]
	Teacher behaviors and student behaviors	Teachers: monitoring, discussion Students: sleeping, working, unengaged, using cell phone, discussion	[96]
		Teacher: explain questions, pointing to the projection, no hand gestures, gesture with both hands, head down and operate, walk around, blackboard writing, guide to raise hand Student: look up, head drop, hand raise, stand up, lying on the desk	[50]
		Teacher: explanation, writing, silence, looking, media display Student: listening, turn, speaking, raising hands, discussion, writing, reading	[49]
		Teacher: guiding Student: writing, reading, listening, turning around, raising hand, standing, discussing	[97]
		Teacher: guiding Student: writing, reading, listening, raising hand, turning around, standing, discussing	[98]
		Teacher: explaining the subject, hitting, holding books, holding cell phone, holding stick, sitting on chair, slapping, walking in classroom, writing on board Student: arguing, clapping, eating in classroom, gossip, hand raise, reading book, sitting on desk, sleeping, standing, talking, writing on textbook	[42]
	Teacher behaviors	Bowing to students, pointing to the blackboard, writing on blackboard, cleaning the blackboard, operating the interactive whiteboard, inviting students to answer questions, walking around classroom and operating the realia	[99]
		Positive behaviors: teaching, blackboard writing, showing; negative behaviors: watching blackboard, watching podium; neutral behavior: operating computer	[35]
		Symbolic action, conscious action, indicative action, evaluative action, and adaptive action	[100]
		Pointing to left, pointing to right, and non-pointing	[101]
		Writing on the blackboard, without obvious intention behavior, describing the teaching content, pointing to the teaching content	[102]
Learning engagement (*n* = 21)	Learning engagement levels	High, moderate, low	[37]
		Very inattentive, inattentive, attentive, absent	[52]
		High, moderate, low	[103]
		Engaged, non-engaged	[104]
		Engaged, non-engaged	[81]
		Highly engaged, nominally engaged, not engaged	[105]
		Minimum learning engagement, low learning engagement, high learning engagement, highest learning engagement	[106]
		Low engagement, medium engagement, high engagement	[51]
		Not engaged at all, nominally engaged, engaged in the task, very engaged	[107]
		Positive, negative	[108]
		Serious, good, fair, poor, and other	[109]
		Low engagement, medium engagement, high engagement	[110]
		High, medium, low	[111]
	Cognitive engagement	ICAP framework: (+2, +1, 0, −1, −2) interactive, constructive, active, passive	[55]
		ICAP framework: (+2, +1, 0, −1, −2) interactive, constructive, active, passive	[56]
		ICAP framework: interactive, constructive, active, passive, other	[24]
		ICAP framework: interactive, constructive, active, passive	[11]
		ICAP framework: disengaged, constructive, active, passive, interactive	[25]
		ICAP framework: disengaged, constructive, active, passive, interactive	[13]
	Behavioral and emotional engagement	Low, medium, high	[112]
		Head posture: nine directions; facial expression: positive emotions, negative emotions	[79]
Attention (*n* = 10)	Attention levels	Attentive, inattentive	[78]
		Low, medium, high	[113]
		Task-unrelated thought, task-related interference; not mind wandering; intentional and unintentional mind wandering	[40]
		Attention, non-attention	[57]
		Engaged, not engaged	[114]
		Focused states, non-concentrating states	[58]
		Dedicated, normal, distracted	[12]
	Attention direction	The direction of the teacher and students’ gaze and body orientation	[21]
		At the teacher, slides, blackboard, taking notes, not concentrated	[2]
		Board, laptop, other, undetected	[38]
Emotion (*n* = 10)	Student emotions	Boredom, confusion, delight, engagement, frustration, and for off-task behavior	[115]
		Happiness, sadness	[116]
		Awe, amusement, confidence, disappointment, neutral	[17]
		Engaged, boredom, neutral	[76]
		Anger, surprise, happiness, neutral, sadness, disgust, fear	[18]
		Active, focused, confused, understanding, depressed, resist, disdain	[45]
		Pleasure, arousal, and dominance: neutral, happiness, sadness, surprise, fear, disgust, anger	[48]
	Teacher emotions	Amusement, awe, confidence, disappointment, neutral	[39]
	Learning task types	Easy, medium, difficult	[29]
		Easy, neutral, difficult	[28]
